# Clinical investigations of receptive and expressive musical functions after stroke

**DOI:** 10.3389/fpsyg.2015.00768

**Published:** 2015-06-12

**Authors:** Ken Rosslau, Daniel Steinwede, C. Schröder, Sibylle C. Herholz, Claudia Lappe, Christian Dobel, Eckart Altenmüller

**Affiliations:** ^1^Clinic of Phoniatrics and Pedaudiology, Muenster University HospitalMuenster, Germany; ^2^Institute of Music Physiology and Musicians‘ Medicine, Hannover University of Music, Drama and MediaHannover, Germany; ^3^Clinic for Neurology, Hannover Medical SchoolHannover, Germany; ^4^German Center for Neurodegenerative Diseases (DZNE)Bonn, Germany; ^5^Institute for Biomagnetism and Biosignalanalysis, University of MuensterMuenster, Germany

**Keywords:** amusia, receptive function, expressive function, music perception, music production

## Abstract

There is a long tradition of investigating various disorders of musical abilities after stroke. These impairments, associated with acquired amusia, can be highly selective, affecting only music perception (i.e., receptive abilities/functions) or expression (music production abilities), and some patients report that these may dramatically influence their emotional state. The aim of this study was to systematically test both the melodic and rhythmic domains of music perception and expression in left- and right-sided stroke patients compared to healthy subjects. Music perception was assessed using rhythmic and melodic discrimination tasks, while tests of expressive function involved the vocal or instrumental reproduction of rhythms and melodies. Our approach revealed deficits in receptive and expressive functions in stroke patients, mediated by musical expertise. Those patients who had experienced a short period of musical training in childhood and adolescence performed better in the receptive and expressive subtests compared to those without any previous musical training. While discrimination of specific musical patterns was unimpaired after a left-sided stroke, patients with a right-sided stroke had worse results for fine melodic and rhythmic analysis. In terms of expressive testing, the most consistent results were obtained from a test that required patients to reproduce sung melodies. This implies that the means of investigating production abilities can impact the identification of deficits.

## Introduction

Although the amusia field is presently dominated by research into congenital cases (e.g., Ayotte et al., 2002), there has been a long tradition of investigations into acquired amusia after stroke or brain damage. We have observed, based on our clinical experience on a stroke unit, that some patients feel that music sounds different to how it did before the stroke. For such cases, we were interested in detecting the characteristics of those deficits as they relate to perceptual and productive musical functions.

Cortical and subcortical brain lesions may cause impairments to such musical functions (Peretz, 1990; Liegeois-Chauvel et al., 1998; Schuppert et al., 2000). These impairments, which manifest themselves clinically in various patterns of deficits in music processing and production (Kohlmetz et al., 2003; DiPietro et al., 2004), are collectively referred to as receptive or expressive amusia. The terms “receptive” and “expressive” are used to represent perceptive and productive musical ability, respectively.

The cognitive processing of music involves high-order neural processing, and it has been claimed that an accurate perception of the melodic and temporal aspects of music requires both “local” and “global” auditory information processing. The terminology of “local” and “global” processing was introduced in the seminal paper by Peretz (1990): In melody perception, the particular interval between two successive notes is assumed to be processed by local, more analytical strategies, whereas the perception of the entire melodic contour requires a more global sense of information processing. The temporal dimension comprises rhythm perception through local strategies and discrimination of meter via global processing mechanisms (Peretz, 1990; see also Liegeois-Chauvel et al., 1998). Those findings were confirmed by our own observations (Schuppert et al., 2000); we detected a more pronounced deficit in local pitch- and rhythm- related ability following left-sided stroke. Conversely, after a right-sided stroke, greater deficits were revealed in global melody contour (use of melodic contextual cues in pitch judgments) and meter processing. Consequently, for this study we retain the systematic view of a differentiation between local and global aspects of melodic and rhythmic processing.

Another key theoretical concept is that of hemispheric specialization for music processing and expression. Previous studies have confirmed the idea of a relative specialization of left and right auditory areas by showing that the left temporal lobe is more involved in the processing of rhythmic, temporal, and sequential features of music than right temporal regions. The latter are more engaged in melodic (pitch and contour) and timbre perception and the perception of spectral features (Samson and Zatorre, 1994; Zatorre and Belin, 2001; Schonwiesner et al., 2005; Lappe et al., 2013). The tendency of melodic and rhythmic processing to lateralize to the left hemisphere in professional musicians has also been demonstrated (Altenmüller, 2001; Russeler et al., 2001; Vuust et al., 2005). Based on the idea of both hemispheres functioning as an intertwined music perception network (Altenmüller, 1989; Schon et al., 2010), an additional bilateral sensitivity to timing information has also been suggested, as well as a higher order system in the superior temporal sulcus, with the processing of slowly modulated signals in the right hemisphere (Boemio et al., 2005). In the case of expressive functions, evidence exists for a differentiation of hemispheric involvement: namely, an asymmetry favoring right temporal regions in the maintenance of pitch while singing (Perry et al., 1999) and in the imagery of tunes (Halpern and Zatorre, 1999; Herholz et al., 2008).

In our view, a lesion study provides an excellent opportunity to clearly associate such receptive and expressive musical functions. The Montreal Battery of Evaluation of Amusia (MBEA; Peretz et al., 2003) is a well-established tool to document incidences of amusia. It contains detailed receptive melodic and metric subtests subdivided into global and local aspects of music processing. However, when we started data collection, the first version of the MBEA seemed to be too complex for use in the clinical routine of a stroke unit. Additionally, the MBEA does not contain any expressive tests, which we considered valuable in addressing the lack of information about expressive musical abilities after a stroke.

In line with the MBEA, one element of the bedside test battery used in this study was a group of five receptive subtests (Pitch Test, Melody Interval Test, Melody Contour Test, Rhythm Test, Meter Test), presented in a previous design to assess melodic and metric music perception by means of discrimination tasks (Schuppert et al., 2000). Additionally, we generated new versions of expressive rhythmic and melodic tests.

**FIGURE 1:**
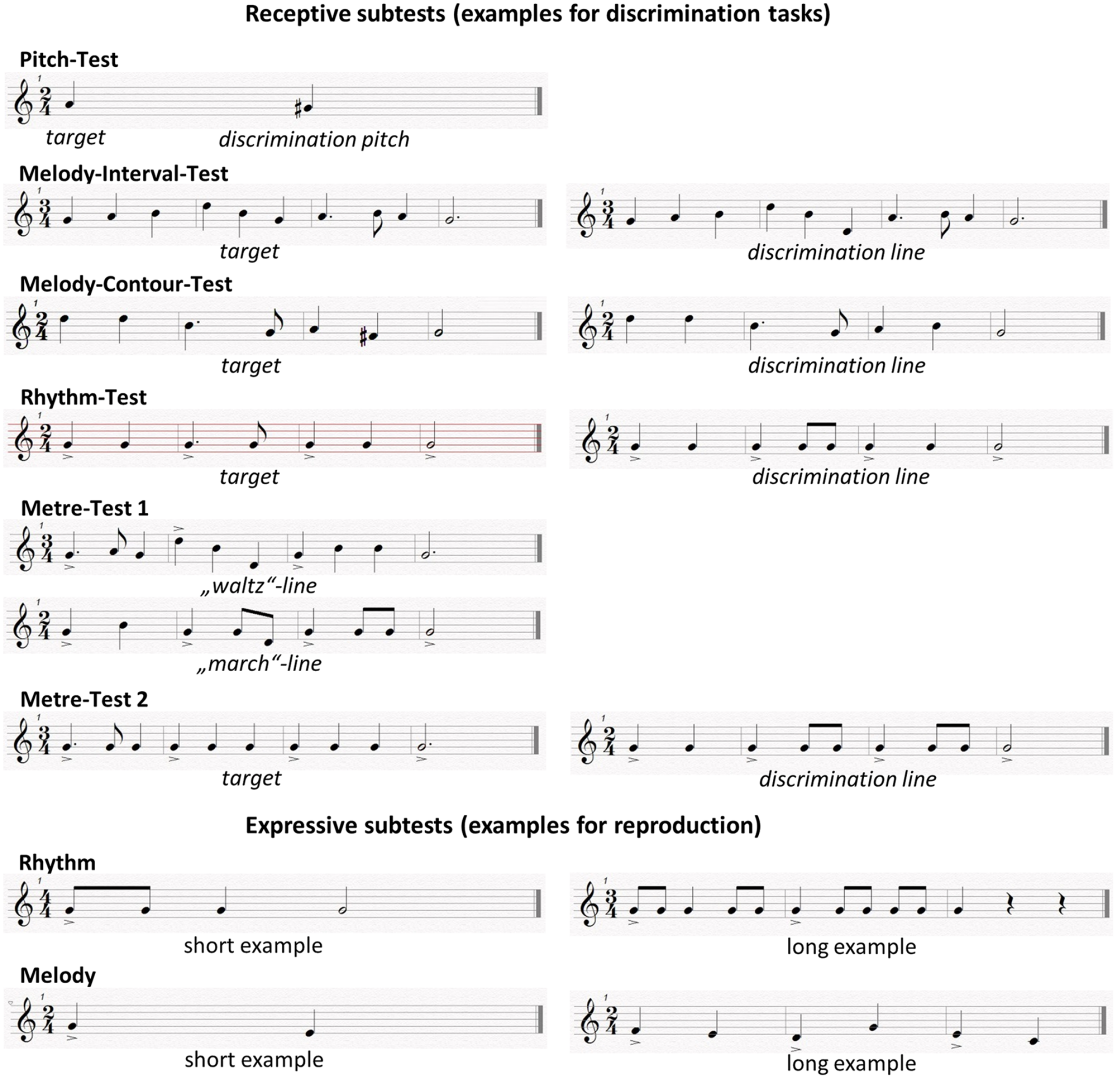
**Examples of receptive and expressive subtests**.

Our study comprises two experiments with separate groups of subjects. After evaluation of the first experiment, we reorganized the receptive and expressive tests for a second experimental setting with some adjustments to better capture the underlying receptive or expressive mechanisms. For the original Meter Test 1, melody sequences had to be identified as a “waltz” or a “marching” meter. The idea was to make it easy to decide whether there was a metric violation or not. However, after evaluation of the first experiment we changed the test to a discrimination task requiring “same”/“different” judgments in line with the other receptive subtests (**Figure 1**), because some subjects were unfamiliar with the musical terms of “waltz” or “marching” meter. The second adjustment concerned the instrument used for the expressive melody test. For the first version of expressive melody testing, we used a xylophone because this instrument is used in early music education at German schools, and it seemed to be appropriate for the simple reproduction of short melody lines. But again after the evaluation of the first experiment, we recognized that the use of a xylophone was much more complex than had been assumed, based on the statements of our subjects complaining about too complex motoric interaction. Due to the fact that Germans also receive a vocal education in school and that good vocal imitation via singing had already been shown for congenital amusics (Tremblay-Champoux et al., 2010), we decided to change this test to one of vocal reproduction.

Creating a direct interaction between the subjects and the investigator was important for the expressive testing of our study. Recent literature has shown that the brain’s mirror neuron system is strongly involved in motoric imitation as well as in vocal imitation, a phenomenon first investigated in song birds (Prather, 2013) that learn their melody lines by imitation. On the basis of various neuroimaging studies, the claim could be made that the intertwined involvement of receptive and expressive functions, especially during vocal production, is a sign of a refined learning system of human social communication (Brown et al., 2004; Ramachandra et al., 2009; Leveque et al., 2013). Recent findings discussed the underlying pathways for vocal and non-vocal perception and production (Loui et al., 2009) which could be interpreted as a reason for a potentially different outcome for instrumental versus vocal imitation. Nevertheless, good results of live vocal interaction during stroke rehabilitation have been obtained with Melodic Intonation Therapy in which patients actively reproduce vocally adapted intonation lines to improve language recovery (Norton et al., 2009).

This study was designed as a straightforward assessment of receptive and especially expressive musical functions that could feasibly be applied in clinical practice. Our main focus was to compare the music perception and production abilities of patients directly after stroke, separately by side of lesion, with those of healthy subjects. Our tests covered the spectral and temporal domains; some focused on a more detailed analysis of musical patterns (Pitch Test, Melody Interval Test, Rhythm Test) and others on a more global music perception ability (Melody Contour Test, Meter Test), on the basis of previous literature on acquired amusia.

In line with previous research, we expected stroke patients to perform significantly worse on receptive and expressive musical tasks than the controls. Furthermore, we expected that on comparing the outcome of the different receptive subtests, local versus global processing would be differentially affected by the side on which the stroke had been experienced. We anticipated greater reduction in local musical function in the Melody Interval Test and the Rhythm Test following a left-sided stroke and a more strongly impaired global musical function in the Melody Contour Test and the Meter Test following a right-sided stroke. For both receptive and expressive musical functions, we hypothesized that musical impairment would depend on the level of musical education that was gained prior to the stroke.

## Materials and Methods

### Subjects

In total, 34 stroke patients (mean age: 57.9) who suffered their first unilateral focal brain lesion in the middle cerebral artery region (15 left-sided, 19 right-sided) and 34 controls, matched for gender, age, degree of musical education and social variables, were included in the two groups of two different experiments (**Table 1**). Patients were recruited from the neurology department of the Hannover Medical School and Minden Hospital.

**Table 1:** Individual data of patients (Age: years; Side and locus of lesion: Left (left-sided), Right (right-sided), STG (superior temporal gyrus), MTG (middle temporal gyrus), ITG (inferior temporal gyrus); Musical education: No (no musical education except in school), Yes (previous musical training instrumental or vocal)).

Participant	Age	Gender	Side and locus of lesion	Musical education
**First Experiment**
P1	51	Female	Left STG/MTG/ITG	No
P2	64	Male	Right STG	No
P3	61	Female	Right STG/MTG	No
P4	60	Male	Left STG/MTG/ITG	Yes
P5	62	Male	Left STG/MTG/ITG	Yes
P6	16	Female	Right STG/MTG	Yes
P7	66	Male	Right MTG	Yes
P8	54	Female	Right STG/MTG	No
P9	60	Male	Right STG	No
P10	56	Male	Right STG/MTG	No
P11	43	Female	Right STG	No
P12	38	Male	Right STG/MTG	No
P13	72	Male	Left MTG/ITG	No
P14	59	Male	Left STG	No
P15	49	Male	Left STG/MTG	No
P16	64	Female	Left STG/MTG	Yes
P17	36	Male	Right STG	Yes
P18	71	Male	Left STG/MTG	Yes
P19	71	Male	Left STG/MTG	Yes
P20	48	Female	Right MTG	Yes
**Second Experiment**
P21	62	Male	Right STG/MTG	No
P22	60	Male	Left STG/MTG/ITG	No
P23	73	Male	Left MTG	No
P24	61	Male	Right MTG/ITG	No
P25	71	Female	Right MTG	No
P26	87	Male	Right STG	Yes
P27	68	Female	Left STG/MTG/ITG	Yes
P28	77	Male	Left MTG/ITG	Yes
P29	27	Female	Right STG	No
P30	73	Female	Left STG/MTG	No
P31	40	Female	Right MTG/ITG	Yes
P32	64	Female	Left STG/MTG/ITG	Yes
P33	50	Female	Right STG	Yes
P34	57	Male	Right STG/MTG	Yes

The main inclusion criteria for the experimental group were that a stroke could be diagnosed on the basis of CT-Scans, that the patient had not had any previous strokes, and that the single ischaemic locus was in the right or left temporal lobe only (i.e., only one affected hemisphere). Patients suffering from large haemorrhagic or ischemic lesions and localizations in subcortical areas were excluded. Patients with additional neurological diseases, hearing impairments, and attention deficits were excluded after clinical examination, as were patients with major movement impairments. Consequently, all patients included in the study were not severely affected by their stroke. Aphasic patients were also excluded in order to ensure a reasonably good vocal imitation ability among the subjects. The assessment of motor functions and speech functions was performed by a neurologist during clinical examination on a stroke unit, according to standard neurologic diagnostics without any additional validated test material; no motor deficits were noted by the neurologist. Attention deficits were assessed by selected subtests (alertness, divided attention, and Go/No Go) of the computerized test battery for attention disorders by Zimmermann and Fimm (1993). All subjects were right-handed and matched with respect to their musical training and general education (Oldfield, 1969). In our study, subjects considered to be musically educated (musically trained) had received instrumental or vocal training of 3–7 years (mean: 4.2 years) in addition to their compulsory school lessons. This meant that they had taken private music lessons and obtained a good level of musicianship, but not that of a professional, or semi-professional musician.

Because of rapid post-lesional reorganization, receptive and expressive musical functions were assessed between the third and the seventh day post-lesion in the form of a bedside test.

The study was conducted in line with the Declaration of Helsinki and approved by the local ethical committee of the Hannover Medical School. All subjects gave written informed consent to participate in the study and were allowed to take pauses or abort the testing at any time.

### Stimulus Material

We used receptive tests that had previously been piloted (Schuppert et al., 2000). The study was conducted in two parts with two different groups of subjects and two experiments. After evaluation of the first group, we adapted the receptive metric subtest and the expressive melodic testing for a second experiment (see Experiments 1 and 2).

#### Receptive Subtests

The receptive subtests (Melody Interval Test, Melody Contour Test, Rhythm Test, and an additional simple Pitch Test) required subjects to compare two short melodic or rhythmic patterns organized in two successive phrases, presented as MIDI-controlled piano sounds (a MIDI sequencing program was used in order to control precise values of pitch, duration, intensity, and velocity). Following the presentation of a target sequence, the original sequence was repeated either exactly the same or with an alteration of one of the following parameters: pitch, contour, rhythm (**Figure 1**). Subjects had to decide whether the second sequence was an identical repetition of the original sequence (i.e., without alteration), or whether it was different from the original (i.e., that one of the above parameters had been altered, although subjects did not need to identify which parameter had been changed). For each receptive subtest, we composed 18 pairs of stimuli. Two thirds (12) of the pairs contained altered sequences in the second sequence of the pair. In the cases of the Melody Interval Test and Rhythm Test, the distinction between the first and second sequence was a pitch change or a change in rhythmic pattern. According to Peretz (1990), these changes required a “local” analysis of music (**Figure 1**).

#### Expressive Subtests

In addition to the receptive testing, two newly developed expressive tests targeted the melodic and rhythmic competences of subjects. For the Expressive Rhythm Test, all subjects had to reproduce 18 short rhythmic patterns, which were presented by the investigator, using wooden sticks. The patterns were in 3/4 or 4/4 m, with full, 1/4 or 1/8 tone length, and consisted of no more than eight beats (ca. 104 beats per min). Expressive melodic function was tested in Experiment 1 (Expressive Melody Test 1) by the reproduction of xylophone phrases and in Experiment 2 (Expressive Melody Test 2) by the reproduction of short sung phrases.

Both the original melody played or sung by the investigator and the subjects’ reproductions were recorded on a DAT-Recorder (**Figure 1**). Subjects were encouraged to reproduce the melodies on a xylophone, after familiarizing themselves with the order of the keys. Playing the xylophone is a very common activity in the German education system, especially in kindergarten. Older adults used to play the xylophone in ensembles with Orff-instruments in kindergarten and primary schools. Furthermore, the keys of the xylophone used in our study were simplified, and specific keys were removed in order to facilitate the reproduction of the melody. In essence, patients had to decide whether a melody was rising or falling and whether there was a turning point. Larger intervals requiring precise representation of distant pitches were avoided.

Singing is also a focus of German music education; especially the older generation participated in group activities in kindergarten and primary school that involved singing. However, as with other musical activities, these were often abandoned later in life.

#### Experiment 1

Data collection began in the first experiment with a group of 20 patients (11 right-sided lesions/9 left-sided lesions; 13 male/7 female) and 20 matched controls, with nine musically educated patients/controls, (termed “Musicians” in this study), and 11 musical novices, (termed “Non-Musicians”). The experiment included the Pitch Test, Melody Interval Test, Melody Contour Test, Rhythm Test and Meter Test 1. For this meter test, 18 melody sequences (half of them presented in a 2/4 and half in a 3/4 m) had to be identified as a “waltz” or a “march.” The original idea was to make it easy to decide whether there was a metric violation or not.

The Expressive Rhythm Test was performed as described in the previous section. For the Expressive Melody Test 1, subjects had to repeat 18 short melody excerpts on a xylophone. The phrases consisted of quarter notes, ranging from C4–C5, at about 60 b.p.m., and consisted of no more than six pitches, with the largest interval between two tones being a fifth (seven semitones).

#### Experiment 2

In the experiment, 14 patients (eight right-sided lesions/six left-sided lesions; seven male/seven female) and 14 matched controls were included, with each group having seven musically educated and seven non-musically educated subjects.

As in the first experiment, the presentation of the receptive subtests (Pitch Test, Melody Interval Test, Melody Contour Test, Rhythm Test) was followed by a Meter Test 2 with some modifications to the first experiment. Because of poor performance in Meter Test 1 (see Results), Meter Test 2 was designed to be more similar to the other subtests in terms of the responses required. It was changed from a meter-identification task to a discrimination task of 18 metric sequences (half in a 2/4 and half in a 3/4 m) on a single pitch (**Figure 1**). In the first experiment, we noticed subjects were confused about how to discriminate between a “march” or a “waltz.” The Expressive Rhythm Test was performed in the same way as in the first experiment. In a second adaption to the first setting, for the Expressive Melody Test 2 subjects had to repeat 18 short melody excerpts sung by the investigator. The melody was composed in the middle range of an untrained voice in a very simple structure corresponding to the melodic xylophone test in the first experiment. Again, melodies consisted of quarter notes within the range of one octave at about 60 b.p.m. Melodies consisted of no more than six pitches, and the largest interval between two tones was a fifth. The test sessions, which consisted of presentation of sung melodies, were performed by the author KR, who is a professionally trained singer. For male subjects, the examples were presented in chest voice and for female subjects, in falsetto voice. Thus, the examples were presented with a voice quality that was similar to the subjects’ voices, an aspect which was considered important for the outcome (for female subjects, the phrase used the fundamental frequency and the interval of a fifth around C4; for male subjects, the fundamental frequency and fifth around C3).

### Procedure

The subjects were tested individually in single sessions in a quiet room. As the technical equipment was very simple, testing could be performed at the bedside during a normal hospital routine. The receptive tests were played with a DAT-Recorder (Sony), using high quality headphones (Sennheiser, dual 2b). The five receptive subtests were presented in randomized order, and the follow-up of the 18 items for each test was pseudo-randomized to avoid systematic effects. Pitch discrimination was, however, always tested first in order to rule out impairments of basic musical function. Each receptive discrimination subtest was preceded by two practice trials. Each new stimulus was introduced by a warning signal. Judgment on each musical stimulus had to be done in a “same–different” classification. Testing could be interrupted at any time at the subject’s request if he or she required a pause.

For rhythmic and melodic expressive testing, the original tunes presented by the investigator and the reproductions of the subjects were recorded with the same DAT-Recorder as was used previously for receptive testing. The administration of the complete receptive and expressive test battery took about 50 min.

### Data Analysis

The results of the receptive subtests, represented by the means of correct answers (hits), and the results of the expressive subtests (score-points of three different raters) were analyzed separately because of different maximum values. The recordings of the expressive rhythmic and melodic subtests were rated by three professional music teachers, who were instructed to pay special attention to the rhythmic and melodic accuracy of patients’ reproductions. For this purpose, these raters had to score the correctness of the imitated rhythmic and melodic sequences on a five-point-scale, blinded as to the group membership of the subjects (**Table 2**). We introduced this scale, which was developed together with our raters, in order to classify the performance of the subjects in a straightforward way. It is important to note that our raters became familiar with each other’s ratings during pilot testing. An inter-rater reliability analysis was performed using the Kappa statistic to determine consistency among raters.

**Table 2:** Rating scale for imitated rhythmic and melodic sequences.

Point-level	Criteria for rhythmic rating	Criteria for melodic rating
4	Complete correspondence to the original line	Complete correspondence to the original line
3	Only small rhythmic mistakes, Good imitation	Only small interval mistakes, Good imitation
2	Problems with rhythmic imitation	Problems with melodic imitation
1	Little correspondence concerning rhythmic correctness	Little correspondence concerning melodic/interval correctness
0	No correspondence to the original line	No correspondence to the original line

The criteria for the scoring were the number of correctly repeated tones and the quantity of rhythmic or melodic-interval errors compared with the version the investigator had presented. The sum-scores were analyzed.

For each receptive and expressive subtest, performance was computed as the percentage of number of correct answers relative to the total number of items. For statistical analyses, the natural logarithm (ln) of the values was used. For descriptive purposes, we used the percentage of correct answers. We performed uni- and multivariate analyses of variance (ANOVA) with factors and covariates as described for each of the results. In all tests, the threshold for statistical significance was set at α = 0.05.

## Results

### Receptive Subtests

#### Comparison of Patients and Controls for the Receptive Tests

We first tested whether patients performed differently from the controls in the receptive subtests of the test battery by using a multivariate ANOVA with factors Status (patient, control) and Musical Education (educated, not educated), and with Experiment (first setting, second setting) as a covariate because data from the two experiments were pooled. With the exception of one subtest (Melody Contour: *F*(1,63) = 2.424, *p* = 0.124), patients performed worse than controls in all subtests [Pitch: *F*(1,63) = 30.039, *p* < 0.001; Melody Interval: *F*(1,63) = 6.990, *p* = 0.010; Rhythm: *F*(1,63) = 26.251, *p* < 0.001; Meter: *F*(1,63) = 10.337, *p* = 0.002]. Further testing revealed that whereas the Melody Contour Test did not differentiate between groups in the first study [Experiment 1, *t*(38) = 0.324, *p* = 0.748], there was a significant group effect in the second study [Experiment 2: *t*(26) = 2.072, *p* = 0.048], with lower scores for patients (**Table 3**).

**Table 3:** Mean values (%) of correct answers for receptive tests (± one standard deviation).

	Experiment 1		Experiment 2	
	Patients (*n* = 20)	Controls (*n* = 20)	Patients (*n* = 14)	Controls (*n* = 14)
Pitch Test	71.1 (21.4)	91.1 (10.4)	80.5 (16.8)	94.8 (9.1)
Melody Interval Test	68.3 (18.8)	75.2 (12.7)	69.4 (17.3)	80.1 (7.1)
Melody Contour Test	72.5 (13.7)	73.1 (9.6)	69.4 (10.6)	76.6 (7.9)
Rhythm Test	64.2 (15.1)	81.7 (16.3)	71.8 (10.3)	89.7 (8.1)
Meter Test 1	50 (16.9)	63.6 (16.9)		
Meter Test 2			62.7 (12.2)	71.8 (10.6)

#### Influence of Musical Education on the Outcome of the Receptive Subtests

In the multivariate ANOVA (MANOVA) described in the section above, musically educated participants performed significantly better than musically non-educated participants in all receptive subtests in both experimental settings [Pitch: *F*(1,63) = 29.25, *p* < 0.001; Melody Interval: *F*(1,63) = 8.58, *p* < 0.001; Melody Contour: *F*(1,63) = 18.73, *p* < 0.001; Rhythm: *F*(1,63) = 14.03, *p* = 0.001; Meter: *F*(1,63) = 9.41, *p* = 0.003]. Furthermore, we investigated the influence of previous musical education on test performance specifically in the subgroup of patients using a multivariate ANOVA in order to make sure that the effect of musical experience also held true for the subgroup of patients. Music Education was used as a factor and Experiment (first, second) as a covariate.

Musically educated patients performed significantly better in all receptive subtests than those not musically educated [Pitch: *F*(1,31) = 21.536, *p* < 0.001; Melody Interval: *F*(1,31) = 8.493, *p* = 0.007; Melody Contour: *F*(1,31) = 5.158, *p* = 0.030; Rhythm: *F*(1,31) = 6.682, *p* = 0.015; Meter: *F*(1,31) = 6.143, *p* = 0.019].

#### Comparison of Receptive Meter Test 1 and Meter Test 2

**FIGURE 2:**
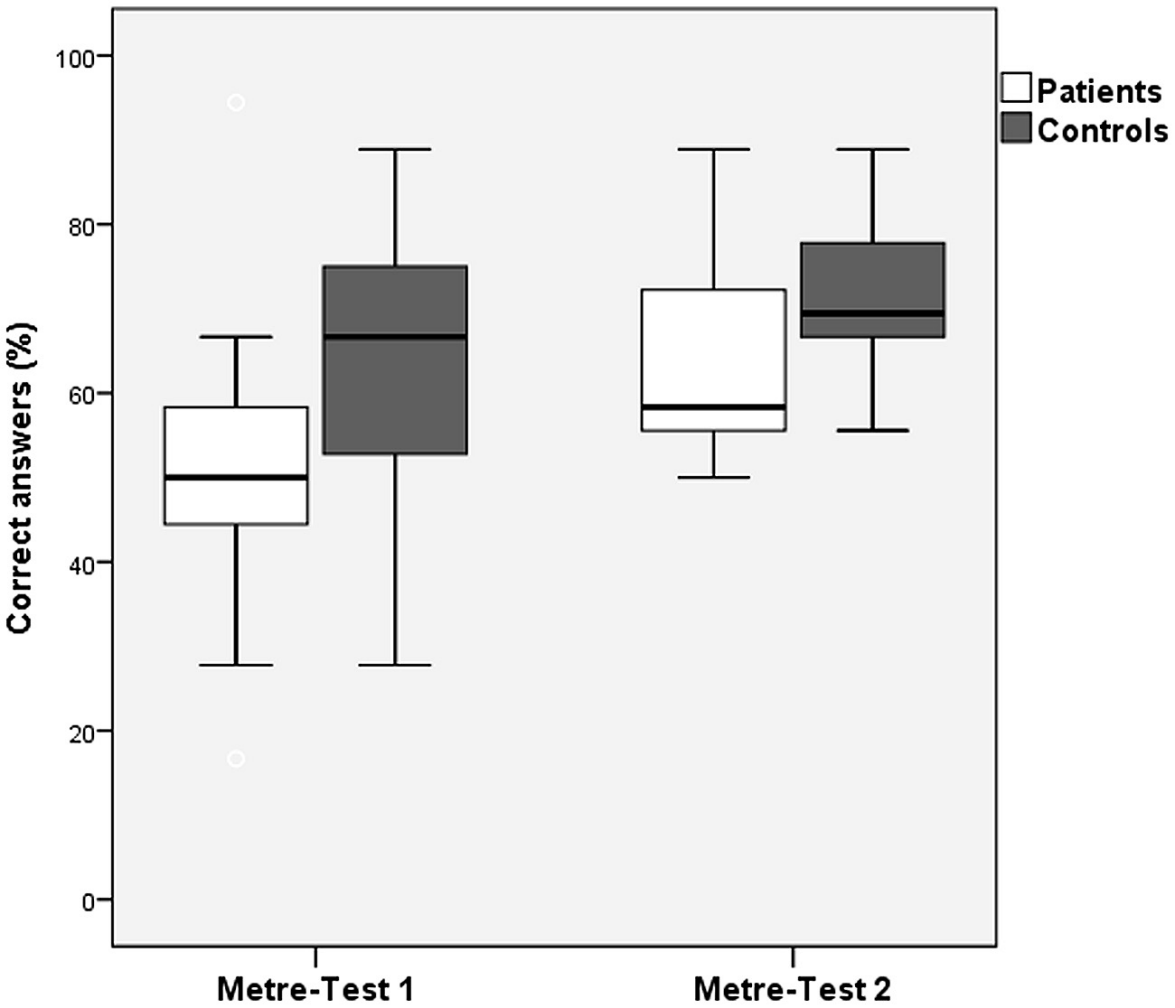
**Correct answers (mean values) of patients and controls for Metre Test 1 vs. Metre Test 2 (error bars indicate one standard deviation)**.

The Meter Test was slightly changed in Experiment 2 compared with Experiment 1. More specifically, the task was to differentiate between a waltz and marching meter in the older version (Experiment 1) and to indicate whether two melodies had the same or a different meter in the newer version (Experiment 2). We tested whether the performance in Meter Test 1 was significantly different from the performance in Meter Test 2. A two-way ANOVA with factors Status (patient, control) and Experiment (Meter Test 1, Meter Test 2) yielded significant main effects of Status [*F*(1,64) = 8.271, *p* = 0.005] and of Experiment [*F*(1,64) = 8.926, *p* = 0.004], with patients performing worse than controls. In the second experiment, patients performed better in the newer Meter Test 2 than those who had participated in the first experiment with the older Meter Test 1. The interaction effect was non-significant, indicating that the performance gain in the newer version of the Meter Test was comparable for patients and controls (**Figure 2**).

#### Influence of the Side of Lesion on the Outcome of Receptive Tests

**FIGURE 3:**
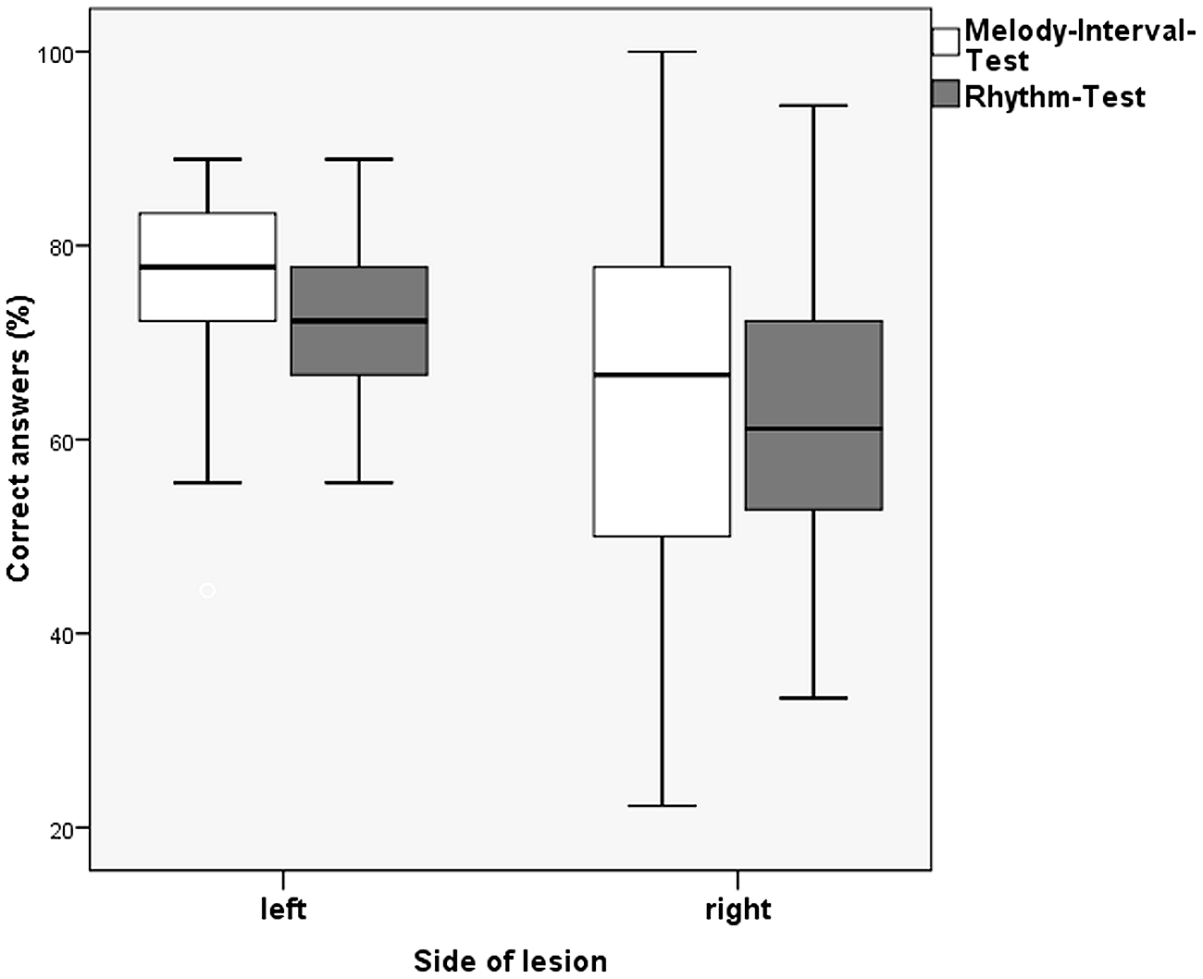
**Correct answers (mean values) of left- and right-sided stroke patients for Melody Interval Test vs. Rhythm Test (error bars indicate one standard deviation)**.

Within the group of patients and across experiments, we analyzed whether the side of the lesion affected performance on the receptive tests by using a multivariate ANOVA with Lesion Side as a factor (left, right) and Experiment (first, second) as a covariate. There were significant main effects of Lesion Side for the Melody Interval Test [*F*(1,31) = 4.254, *p* = 0.048], and for the Rhythm Test [*F*(1,31) = 6.178, *p* = 0.019], with left-hemispheric lesion patients performing better than right-hemispheric lesion patients in both tests and with no significant difference between the experiments. The main effects of Lesion Side were not significant for the Pitch, Melody Contour, and Meter Tests (**Figure 3**).

### Expressive Subtests

#### Comparison of Patients and Controls on the Expressive Tests

The inter-rater reliability showed a substantial agreement in all expressive subtests for both patients and controls (kappa for each subtest ranged between 0.62–0.78; *p* < 0.001). For the expressive subtests, we first tested whether patients performed significantly differently from controls using a multivariate ANOVA with the factors Status (patient, control) and Musical Education (educated, non-educated) and the covariate Experiment (first setting, second setting; **Table 4**). For both subtests, the main effect of Status was significant, with patients performing worse than controls in both experimental settings [Expressive Rhythm Test: *F*(1,63) = 38.535, *p* < 0.001; Expressive Melody Test: *F*(1,63) = 7.042, *p* = 0.010].

**Table 4:** Mean values (%) of correct answers for expressive tests (± one standard deviation).

	Experiment 1		Experiment 2	
	Patients	Controls	Patients	Controls
Expressive Rhythm Test	75.2 (8.7)	86.1 (6.1)	71.2 (8.1)	83.5 (9.1)
Expressive Melody Test 1	69.9 (11.7)	73.6 (9.6)		
Expressive Melody Test 2			75.2 (12.2)	85.2 (12.3)

#### Influence of Musical Education on the Outcome of the Expressive Subtests

**FIGURE 4:**
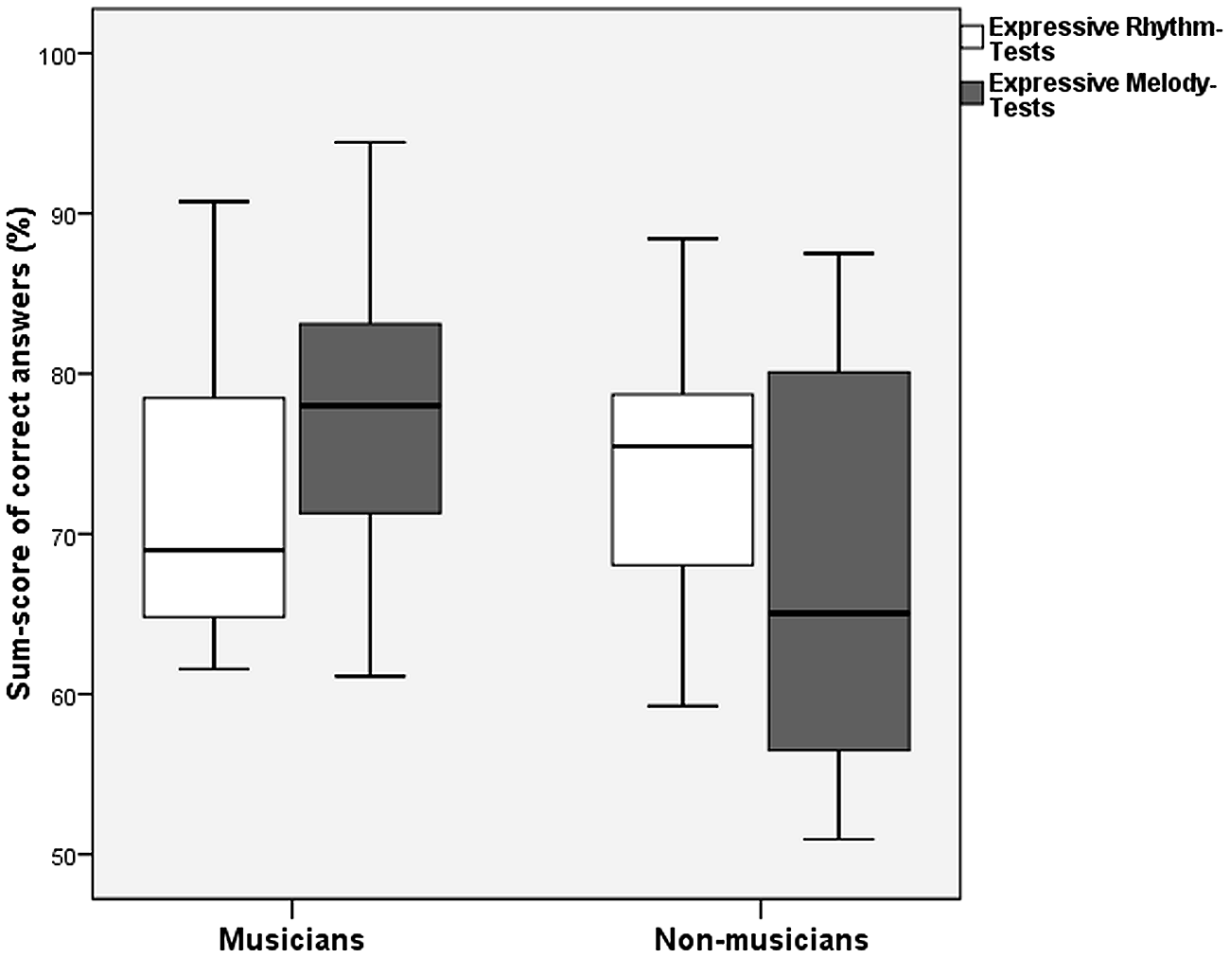
**Sum scores of patients with musical education and patients without for Expressive Rhythm Tests vs. Expressive Melody Tests (error bars indicate one standard deviation)**.

In the multivariate ANOVA described in the previous paragraph, musically educated participants performed significantly better than musically non-educated participants in the Expressive Melody Tests in both experiments [*F*(1,63) = 28.44, *p* < 0.001]; however, there was no significant influence of musical education on the Expressive Rhythm Test [*F*(1,65) = 1.76, *p* = 0.189; **Figure 4**]. The interaction of Musical Education and Status was significant only for the Expressive Rhythm Test [*F*(1,63) = 16.17, *p* = 0.017]. In order to test whether there was an effect of Musical Education for the subgroup of patients, we performed a multivariate ANOVA on patients only (factor Musical Education, covariate Experiment). Musically educated patients outperformed musically non-educated patients significantly again only in the Expressive Melody Tests, but there was no difference in the Expressive Rhythm Tests [Expressive Melody Tests *F*(1,31) = 7.64, *p* = 0.010; Expressive Rhythm Tests *F*(1,31) = 0.046, *p* = 0.505].

#### Influence of the Side of Lesion on the Outcome of Expressive Tests

To answer the question of whether the lesion side affected performance on the rhythmic and melodic expressive subtests, we conducted a multivariate ANOVA on the group of patients with factor Lesion side (left, right) and Experiment (first, second) as covariate. Lesion side did not significantly affect performance on the expressive rhythmical and melodic subtests [Expressive Rhythm Test: *F*(1,31) = 1.50, *p* = 0.230; Expressive Melody Test 1 and 2: *F*(1,31) = 3.44, *p* = 0.077].

#### Expressive Testing via Singing Compared to Eexpressive Testing via Xylophone Use

**FIGURE 5:**
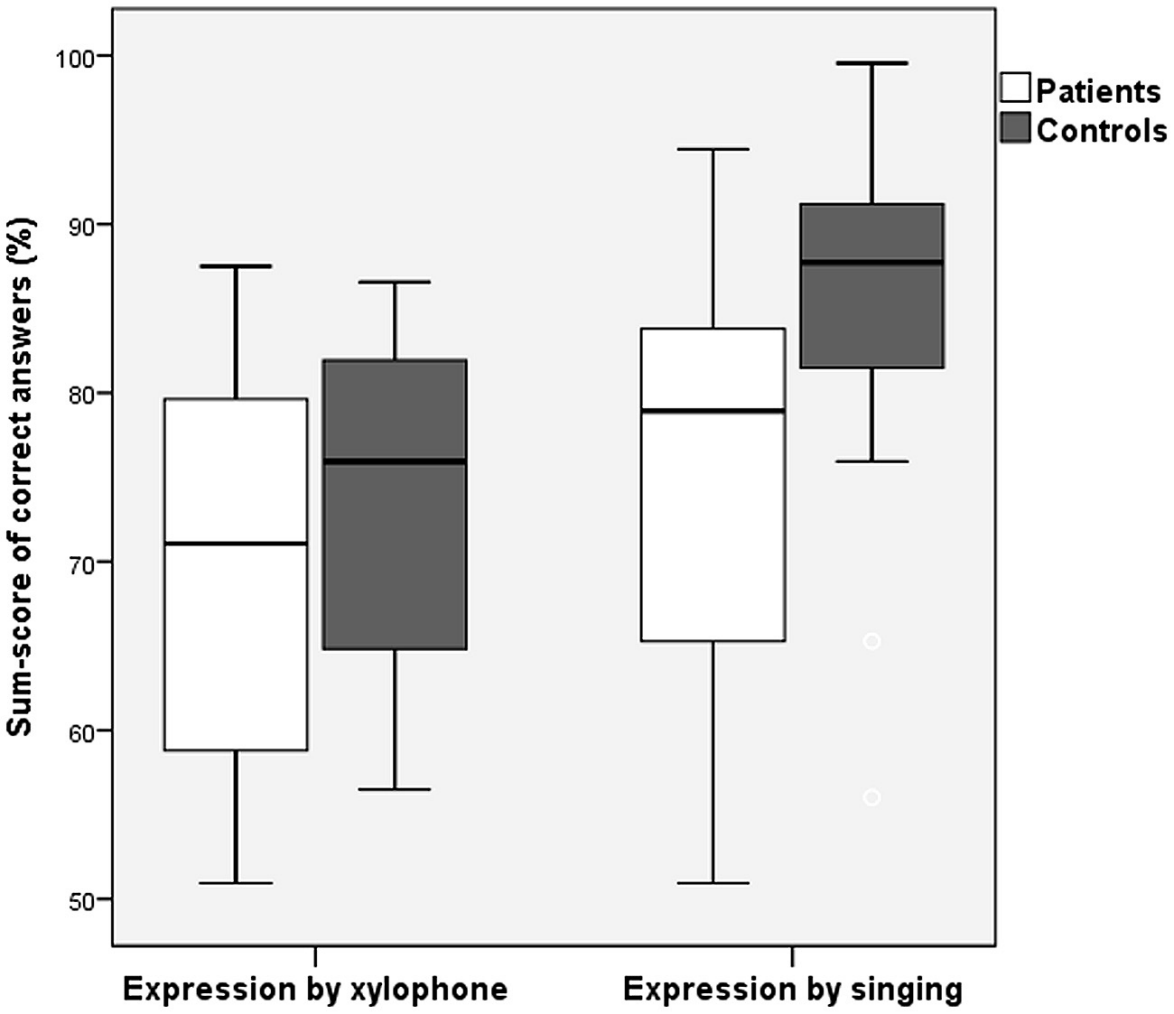
**Sum scores of patients and controls for expressive production by xylophone vs. singing (error bars indicate one standard deviation)**.

We were further interested if the new version of the Expressive Melody Test (singing compared to the xylophone used in the previous version) led to better performance. To this end, we computed a univariate ANOVA with factors Status (patient, control) and Experiment (Expressive Melody Test 1, Expressive Melody Test 2). Significant main effects of Status [*F*(1,64) = 5.360, *p* = 0.024] and of Experiment [*F*(1,64) = 7.474, *p* = 0.008] indicated that the patients performed worse than the controls, but that overall performance was better in the new singing version (**Figure 5**). The interaction of Status and Experiment was not significant, indicating that the performance gain in the new test version was comparable for both groups.

## Discussion

The idea for the present study resulted from interviews prior to testing where some patients complained about having a different musical impression of a previously well-known song, compared to their experience prior to stroke. The main aim of the study was to compare the receptive and expressive musical domain across left- and right-lesioned patients and healthy subjects.

In contrast to the test battery that is offered by the MBEA (Peretz et al., 2003) that only includes receptive test, we combined both receptive and expressive rhythmic and melodic tests. To date, there is a lack of knowledge concerning expressive musical functions after stroke. Here, we provide a detailed picture of deficits by systematically testing a total of 34 stroke patients and their matched controls. We are convinced that it is useful to assess amusic deficits in a narrow time window between Post-lesional Day 3 and 7, since deficits seem to be most obvious in the first 2 weeks following a stroke. Later, brain reorganization and neuroplastic effects obscure the primary deficits. This is one of the reasons why, in a related study, recovery from amusia was a common finding (Schuppert et al., 2003). To investigate the dependence of musical perception and expression on the temporal auditory areas, we decided to include only lesions with a focused area in the temporal lobe in one hemisphere. As a result, our patients were less affected by motor impairments, aphasia, or attentional deficits than other stroke patients, and were therefore better able to complete the complex reproduction tasks in the expressive testing section. Furthermore, we were interested in presenting the test material in line with recent literature with a focus on detailed local analysis (Pitch Test, Rhythm Test and Melody Interval Test) and on holistic global analysis (Melody Contour Test, Meter Test) of melodic and metric elements. Consequently, we were able to investigate the effects of brain lateralization, as discussed in previous literature (Peretz, 1990; Liegeois-Chauvel et al., 1998; Peretz et al., 2003).

### Discussion of the Results

A notable effect in nearly all of the receptive and expressive subtests was that stroke patients with the above-described lesions in the left or right temporal regions showed significant impairments in both receptive and expressive modalities compared with healthy control subjects.

The side of the lesion had a significant effect on the receptive Melody Interval Test and the Rhythm Test, with left-hemispheric lesioned patients performing better than right-hemispheric lesioned patients in both tests. This contradicts the idea of a primarily left-sided processing of local information, such as interval and rhythm perception (Peretz, 1990; Liegeois-Chauvel et al., 1998). The evaluation and comparison of the other subtests failed to demonstrate a clear relation of the site of lesion to either local functions (hypothesized after left-sided stroke) or global functions (hypothesized after right sided stroke). Until now, lesion studies investigating local and global processing separately have also failed to clearly demonstrate such a dichotomy (Schuppert et al., 2000). Consequently, our results are in line with studies demonstrating music processing based on intertwined networks in both hemispheres (Gordon et al., 2010; Schon et al., 2010).

Although we had assumed that the instruction to choose between “waltz” or “march” style after the presentation of a rhythmic sequence in Meter Test 1 was appropriate for testing meter perception, performance was poor in this test, possibly due to difficulty understanding the instructions. This was corroborated by reported confusion of the subjects about interpreting a “waltz” or a “marching” style. Consequently, we aligned the metric test to the other receptive subtests by changing it to one of discrimination between two rhythmic sequences (either 3/4 or 2/4 m). We interpret the significantly raised mean level of performance of Meter Test 2 as the consequence of a more specific testing of the metric domain. Better understanding of instructions and reduced misinterpretation due to lack of knowledge of music styles probably contributed to the improved performance.

### Effect of Previous Musical Education

In general, subjects with a basic musical education performed significantly better in all receptive and expressive subtests than those patients without, showing that even a short period of musical education, sometimes decades before the stroke, seems to influence the outcome in receptive and expressive tests. Here, one could argue that our testing battery is not necessarily sensitive to neural damage itself, but is only testing specific musical abilities. However, since we carefully matched our patients with healthy elderly controls sharing the same education and musical background, and since patients performed worse than controls, we do not believe that we merely demonstrate the outcome of a “musicality test.” We agree that it is difficult to claim any long-lasting “protective effects” of previous individual music education; however, we want to underscore that our results may indicate that such an education could have a long-term effect by providing the subjects early in life with multiple representations of music and various music-processing strategies. In a longitudinal study, we demonstrated that different styles of music education had distinct effects on brain networks involved in music processing in adolescents and older music students (Altenmüller et al., 1997, 2000). We therefore speculate that following the destructive effect of a stroke on specific cortical areas, previous musical training might supply alternative, vicarious networks, taking over functions that are important for intact music processing. Relevant in this context are recent findings that early musical training can have long-term effects on cognitive performance (Hanna-Pladdy and MacKay, 2011) and that previous musical training improves vowel perception in elderly people (Bidelman and Alain, 2015). It is important to mention that there is no way to distinguish musical aptitude from abilities generated by musical education.

### Discussion of the Expressive Tests

Assuming the involvement of a receptive pathway during musical expression, it is surprising that patients were not more impaired for expressive than for receptive subtests, compared to controls. In the first experiment, we used a xylophone for expressive melody testing. As mentioned in the method section, playing on a xylophone is, besides singing, a very common musical activity in German schools, so we assumed that the simple reproduction of short sequences in this manner would be easy for our subjects. The low scores in the first experimental setting were surprising and we did not consider them representative of performance in the expressive melodic domain. It prompted us to change the expressive melodic test to the reproduction of the melodies by singing, which we thought would also engage basic melodic function. The results of the melodic testing indicate that both the xylophone and the vocal imitation test version may actually be similarly valid as tests for musical expression, but that the xylophone version taps into more complex abilities, which are also more challenging for the controls. Although both types of melodic reproduction form part of German musical education, there is a clear advantage for vocal expression because it is the basic tool for speech acquisition, and vocal imitation forms part of many activities in everyday life. When playing an instrument, musical interpretation has to be transferred to a motor system that is a priori not necessarily linked to sound production. It can be assumed that vocal imitation provides a closer proxy for internal musical processing due to less complicated and more intuitive production pathways. Therefore, it was not very surprising to observe much better results for the sung expressive melody test as compared with the instrumental reproduction via xylophone. Those different findings for musical expression are in line with findings of separate pathways for pitch perception for vocal or non-vocal stimuli (Loui et al., 2009), which we consider very important for receptive processing after stroke. Nevertheless, the voice is the innate tool for communication and sound production. In this respect, our study underlines those basic mechanisms showing that even highly affected patients are able to express themselves quite well vocally.

Although results concerning receptive and expressive musical functions depended heavily on the musical level of the individual patient, the general ability to actively and accurately reproduce presented melodies was remarkable. We consider this to be a consequence of increased facilitation and motivation during the expressive tests engendered by live interaction with the investigator.

### Further Aspects

Stroke-related amusia frequently remains unnoticed. However, according to our clinical observations this dysfunction seems to have an influence on both receptive and expressive musical functions of patients.

Our main focus for this study was to assess musical abilities in so-called “average patients” – those who have suffered only a slight version of a stroke without any additional deficits – and to compare these to the same functions in healthy controls. To be appropriate for a stroke unit, testing must be time-effective. Consequently, the performance of our battery required a testing time under an hour. Nevertheless, for standardization of a valid diagnostic tool and to investigate the interaction of the lesion side and musicianship it is necessary to increase the group size. Additionally, because of the difficulties of live-interactive testing further investigation is needed in terms of the appropriate standards for live presentation of stimulus. In particular, our protocol requires the investigator to be musically competent (either with an instrument or vocally) in order to administer the expressive subtests.

The evaluation of musical functions is mainly suited to patients with a small lesion, who are less widely affected than those with other stroke-associated deficits of the motor and speech system. Given the near complete recovery of receptive musical deficits (Schuppert et al., 2003), the use of a standardized diagnostic tool during the routine on a stroke unit is questionable. But for a more limited use with those patients complaining about a receptive or expressive musical deficit and especially for professional musicians such a battery could be meaningful. For investigations especially with professional musicians, we would strongly recommend the validated test battery of Schon et al. (2001, 2004).

A further research topic could also be a systematic assessment of such music-related complaints of stroke patients concerning deficits of their “emotional interpretation” of known music pieces in a longitudinal design. In this way one could learn more about the behavioral outcomes of reorganization after a stroke in relation to musical abilities.

## Conflict of Interest Statement

The authors declare that the research was conducted in the absence of any commercial or financial relationships that could be construed as a potential conflict of interest.

## References

[B1] Altenmüller E. (1989). Cortical DC-potentials as electrophysiological correlates of hemispheric dominance of higher cognitive functions. *Int. J. Neurosci.* 47 1–14. 10.3109/00207458908987413 2793333

[B2] Altenmüller E. O. (2001). How Many Music Centers are in the Brain? *Ann. N. Y. Acad. Sci*. 930 273–280. 10.1111/j.1749-6632.2001.tb05738.x 11458834

[B3] Altenmüller E. Bangert M. Liebert G. Gruhn W. (2000). Mozart in Us: how the Brain processes Music. *Med. Probl. Perform. Art.* 15 99–106.

[B4] Altenmüller E. Gruhn W. Parlitz D. Kahrs J. (1997). Music learning produces changes in brain activation patterns: a longitudinal DC-EEG-Study. *Int. J. Arts Med.* 5 28–34.

[B5] Ayotte J. Peretz I. Hyde K. L. (2002). Congenital amusia: a group study of adults aﬄicted with a music-specific disorder. *Brain* 125 238–251. 10.1093/brain/awf028 11844725

[B6] Bidelman G. M. Alain C. (2015). Musical training orchestrates coordinated neuroplasticity in auditory brainstem and cortex to counteract age-related declines in categorical vowel perception. *J. Neurosci.* 35 1240–1249. 10.1523/JNEUROSCI.3292-14.2015 25609638 PMC6605547

[B7] Boemio A. Fromm S. Braun A. Poeppel D. (2005). Hierarchical and asymmetric temporal sensitivity in human auditory cortices. *Nat. Neurosci.* 8 389–395. 10.1038/nn1409 15723061

[B8] Brown S. Martinez M. J. Hodges D. A. Fox P. T. Parsons L. M. (2004). The song system of the human brain. *Brain Res. Cogn. Brain Res.* 20 363–375. 10.1016/j.cogbrainres.2004.03.016 15268914

[B9] DiPietro M. Laganaro M. Leemann M. Schnider A. (2004). Receptive amusia: temporal auditory processing deficit in a professional musician following a left temporo-parietal lesion. *Neuropsychologia* 42 868–877. 10.1016/j.neuropsychologia.2003.12.004 14998702

[B10] Gordon R. L. Schon D. Magne C. Astesano C. Besson M. (2010). Words and melody are intertwined in perception of sung words: EEG and behavioral evidence. *PLoS ONE* 5:e9889 10.1371/journal.pone.0009889 PMC2847603 20360991

[B11] Halpern A. R. Zatorre R. J. (1999). When that tune runs through your head: a PET investigation of auditory imagery for familiar melodies. *Cereb. Cortex* 9 697–704. 10.1093/cercor/9.7.697 10554992

[B12] Hanna-Pladdy B. MacKay A. (2011). The relation between instrumental musical activity and cognitive aging. *Neuropsychology* 25 378–386. 10.1037/a0021895 21463047 PMC4354683

[B13] Herholz S. C. Lappe C. Knief A. Pantev C. (2008). Neural basis of music imagery and the effect of musical expertise. *Eur. J. Neurosci.* 28 2352–2360. 10.1111/j.1460-9568.2008.06515.x 19046375

[B14] Kohlmetz C. Muller S. V. Nager W. Munte T. F. Altenmuller E. (2003). Selective loss of timbre perception for keyboard and percussion instruments following a right temporal lesion. *Neurocase* 9 86–93. 10.1076/neur.9.1.86.14372 16210228

[B15] Lappe C. Steinstrater O. Pantev C. (2013). Rhythmic and melodic deviations in musical sequences recruit different cortical areas for mismatch detection. *Front. Hum. Neurosci.* 7:260 10.3389/fnhum.2013.00260 PMC3675320 23759929

[B16] Leveque Y. Muggleton N. Stewart L. Schon D. (2013). Involvement of the larynx motor area in singing-voice perception: a tms study(dagger). *Front. Psychol.* 4:418 10.3389/fpsyg.2013.00418 PMC3708144 23874314

[B17] Liegeois-Chauvel C. Peretz I. Babai M. Laguitton V. Chauvel P. (1998). Contribution of different cortical areas in the temporal lobes to music processing. *Brain* 121(Pt 10), 1853–1867. 10.1093/brain/121.10.1853 9798742

[B18] Loui P. Alsop D. Schlaug G. (2009). Tone deafness: a new disconnection syndrome? *J. Neurosci*. 29 10215–10220. 10.1523/JNEUROSCI.1701-09.2009 19692596 PMC2747525

[B19] Norton A. Zipse L. Marchina S. Schlaug G. (2009). Melodic intonation therapy: shared insights on how it is done and why it might help. *Ann. N. Y. Acad. Sci.* 1169 431–436. 10.1111/j.1749-6632.2009.04859.x 19673819 PMC2780359

[B20] Oldfield R. C. (1969). Handedness in muscians. *Br. J. Psychol.* (London, England: 1953) 60 91–99. 5779028 10.1111/j.2044-8295.1969.tb01181.x

[B21] Peretz I. (1990). Processing of local and global musical information by unilateral brain-damaged patients. *Brain* 113(Pt 4), 1185–1205. 10.1093/brain/113.4.1185 2397389

[B22] Peretz I. Champod A. S. Hyde K. (2003). Varieties of musical disorders. the montreal battery of evaluation of amusia. *Ann. N. Y. Acad. Sci.* 999 58–75. 10.1196/annals.1284.006 14681118

[B23] Perry D. W. Zatorre R. J. Petrides M. Alivisatos B. Meyer E. Evans A. C. (1999). Localization of cerebral activity during simple singing. *Neuroreport* 10 3979–3984. 10.1097/00001756-199912160-00046 10716244

[B24] Prather J. F. (2013). Auditory signal processing in communication: perception and performance of vocal sounds. *Hear. Res.* 305 144–155. 10.1016/j.heares.2013.06.007 23827717 PMC3818290

[B25] Ramachandra V. Depalma N. Lisiewski S. (2009). The role of mirror neurons in processing vocal emotions: evidence from psychophysiological data. *Int. J. Neurosci.* 119 681–690. 10.1080/00207450802572188 19283593

[B26] Russeler J. Altenmuller E. Nager W. Kohlmetz C. Munte T. F. (2001). Event-Related Brain Potentials to Sound Omissions Differ in Musicians and Non-Musicians. *Neurosci. Lett.* 308 33–36. 10.1016/S0304-3940(01)01977-2 11445279

[B27] Samson S. Zatorre R. J. (1994). Contribution of the right temporal lobe to musical timbre discrimination. *Neuropsychologia* 32 231–240. 10.1016/0028-3932(94)90008-6 8190246

[B28] Schon D. Gordon R. Campagne A. Magne C. Astesano C. Anton J. L. (2010). Similar cerebral networks in language, music and song perception. *Neuroimage* 51 450–461. 10.1016/j.neuroimage.2010.02.023 20156575

[B29] Schon D. Lorber B. Spacal M. Semenza C. (2004). A selective deficit in the production of exact musical intervals following right-hemisphere damage. *Cogn. Neuropsychol.* 21 773–784. 10.1080/02643290342000401 21038233

[B30] Schon D. Semenza C. Denes G. (2001). Naming of musical notes: a selective deficit in one musical clef. *Cortex* 37 407–421. 10.1016/S0010-9452(08)70581-1 11485065

[B31] Schonwiesner M. Rubsamen R. von Cramon D. Y. (2005). Hemispheric asymmetry for spectral and temporal processing in the human antero-lateral auditory belt cortex. *Eur. J. Neurosci.* 22 1521–1528. 10.1111/j.1460-9568.2005.04315.x 16190905

[B32] Schuppert M. Münte T. Altenmuller E. (2003). Recovery from receptive amusia suggests functional reorganization of music-processing networks. *J. Neuropsychol.* 14 113–122. 10.1024//1016-264X.14.2.113

[B33] Schuppert M. Munte T. F. Wieringa B. M. Altenmuller E. (2000). Receptive amusia: evidence for cross-hemispheric neural networks underlying music processing strategies. *Brain* 123(Pt 3), 546–559. 10.1093/brain/123.3.546 10686177

[B34] Tremblay-Champoux A. Dalla Bella S. Phillips-Silver J. Lebrun M. A. Peretz I. (2010). Singing proficiency in congenital amusia: imitation helps. *Cogn. Neuropsychol.* 27 463–476. 10.1080/02643294.2011.567258 21864199

[B35] Vuust P. Pallesen K. J. Bailey C. van Zuijen T. L. Gjedde A. Roepstorff A. (2005). To musicians, the message is in the meter pre-attentive neuronal responses to incongruent rhythm are left-lateralized in musicians. *Neuroimage* 24 560–564. 10.1016/j.neuroimage.2004.08.039 15627598

[B36] Zatorre R. J. Belin P. (2001). Spectral and temporal processing in human auditory cortex. *Cereb. Cortex* 11 946–953. 10.1093/cercor/11.10.946 11549617

[B37] Zimmermann P. Fimm B. (1993). *A Computerized Neuropsychological Assessment of Attention Deficits (Manual)*. Herzogenrath: PsyTest.

